# Glutamine deamidation does not increase the immunogenicity of C-peptide in people with type 1 diabetes

**DOI:** 10.1016/j.jtauto.2022.100180

**Published:** 2022-12-27

**Authors:** Abby Foster, Pushpak Bhattacharjee, Eleonora Tresoldi, Miha Pakusch, Fergus J. Cameron, Stuart I. Mannering

**Affiliations:** a Immunology and Diabetes Unit, St. Vincent's Institute of Medical Research, 9 Princes St, Fitzroy, Victoria, 3065, Australia; b Department of Medicine, University of Melbourne, St. Vincent's Hospital, Fitzroy, Victoria, 3065, Australia; c Department of Endocrinology and Diabetes, Royal Children's Hospital, Australia; d Murdoch Children's Research Institute, Parkville, VIC, Australia; e Department of Paediatrics, University of Melbourne, Melbourne, VIC, Australia

**Keywords:** Type 1 diabetes, C-peptide, Deamidation, CD4^+^ T cell, Neoepitope

## Abstract

Type 1 diabetes (T1D) is a T-cell mediated autoimmune disease in which the insulin-producing beta cells are destroyed. While it is clear that full-length C-peptide, derived from proinsulin, is a major antigen in human T1D it is not clear how and why C-peptide becomes a target of the autoimmune CD4^+^ T-cell responses in T1D. Neoepitopes formed by the conversion of glutamine (Q) residues to glutamic acid (E) by deamidation are central to the immune pathogenesis of coeliac disease and have been implicated in autoimmune responses in T1D. Here, we asked if the immunogenicity of full-length C-peptide, which comprises four glutamine residues, was enhanced by deamidation, which we mimicked by substituting glutamic acid for glutamine residue. First, we used a panel of 18 well characterized CD4^+^ T-cell lines specific for epitopes derived from human C-peptide. In all cases, when the substitution fell within the cognate epitope the response was diminished, or in a few cases unchanged. In contrast, when the substitution fell outside the epitope recognized by the TCR responses were unchanged or slightly augmented. Second, we compared CD4^+^ T-cell proliferation responses, against deamidated and unmodified C-peptide, in the peripheral blood of people with or without T1D using the CFSE-based proliferation assay. While, as reported previously, responses were detected to unmodified C-peptide, no deamidated C-peptide was consistently more stimulatory than native C-peptide. Overall responses were weaker to deamidated C-peptide compared to unmodified C-peptide. Hence, we conclude that deamidated C-peptide does not play a role in beta-cell autoimmunity in people with T1D.

## 1 Introduction

Type 1 diabetes (T1D) is a chronic, incurable, autoimmune disease caused by the T-cell mediated destruction of the pancreatic, insulin producing, beta-cells [1]. This leads to insulin deficiency and dysregulation of glucose metabolism [2]. While considerable progress has been made in identifying antigens and epitopes recognized by T cells associated with T1D [3], the full repertoire of epitopes recognized by pathogenic human T cells remain unclear. A detailed knowledge of the epitopes recognized by T1D-associated T cells is required for the rational development of antigen-specific therapies and assays to monitor changes in the number and function of pathogenic T cells in T1D.

Several lines of evidence point at a central role for CD4^+^ T cells in the immune pathogenesis of T1D, including the following. Genome-wide association studies (GWAS) have revealed that the risk of developing T1D is strongly associated with the HLA class II specifically the haplotypes: HLA-DR3-DQ2 (HLA-DRB1*03:01-DQA*05:01-DQB*02:01) and HLA-DR4-DQ8 (HLA-DRB1*04:01-DQA*03:01; DQB*03:02) [4]. Individuals who are heterozygous for HLA-DQ2 and -DQ8 are at greatest risk of developing T1D which is attributed to the formation of DQ2/DQ8 transdimers (*trans*) [5]. CD4^+^ T cells ‘help’ is required for antibody isotype switching. Isotype switched autoantibodies, specific for beta-cell antigens, arise before the clinical onset of T1D, suggesting that CD4^+^ T cells play a direct role [6]. Finally, beta-cell antigen specific CD4^+^ T cells, restricted by T1D-associated HLA allomorphs, infiltrate the pancreatic islets of people with T1D, putting CD4^+^ T cells at the ‘scene of the crime’ [[7], [8], [9]].

Insulin, and its precursor proinsulin, are important targets of autoimmune responses in T1D [10]. C-peptide is a 31-amino acid peptide that is excised from proinsulin during the biosynthesis of insulin [11]. Our analysis of CD4^+^ T cells that infiltrated the residual pancreatic islets of deceased organ donors who suffered from T1D revealed that many islet-infiltrating CD4^+^ T cells were specific for epitopes derived from the C-peptide of proinsulin [7]. Many of these cloned islet-infiltrating CD4^+^ T-cells recognized C-peptide epitopes presented by HLA-DQ8, or HLA-DQ8*trans* [7]. The observation that C-peptide specific CD4^+^ T cells infiltrate human islets in T1D has subsequently been confirmed by others [8,9]. To further evaluate the clinical relevance of human CD4^+^ T-cell responses against C-peptide we asked if CD4^+^ T-cell responses against full-length C-peptide (PI_33-63_) could be detected in the peripheral blood of people with, or without, T1D. We found that C-peptide specific responses could be detected in PBMC samples from over 60% of people with T1D [12]. C-peptide specific clones were isolated from six subjects. Analysis of these clones revealed fourteen distinct C-peptide derived epitopes that were restricted by either: HLA-DR4, -DQ8, -DQ2, DQ8*trans* and DQ2*trans* [12]. More recently, independent studies have confirmed the clinical relevance of CD4^+^ T-cell responses against full-length C-peptide [13]. Hence there is considerable evidence that CD4^+^ T-cell responses play a direct role in the autoimmune pathogenesis of human T1D.

A central question in autoimmunity is: why does the immune system target otherwise healthy tissues? One compelling hypothesis is that the autoimmune response targets, to some extent, self-antigens that have been modified. These modified self-epitopes, known collectively as neoepitopes, could be the targets of autoimmune response in autoimmune diseases [14,15]. This concept has been widely investigated in autoimmunity and has received considerable experimental support in T1D and other autoimmune diseases. In T1D the first neoepitope was reported in 2005 [16], since that time a variety of neoepitopes have been described that are the targets of CD4^+^ T cells in T1D [10,15,17]. Neoepitopes can form by a wide array of mechanisms [14,18]. One of the best characterized examples is the creation of neoepitopes that occurs in coeliac disease where the cereal protein gliadin is modified by transglutaminase which converts glutamine (Q) residues to glutamic acid (E), a process known as deamidation [19,20]. Coeliac disease is an autoimmune disease triggered by responses to dietary gluten [21]. Like T1D, coeliac disease is associated with HLA-DQ2 and DQ8. CD4^+^ T-cell responses associated with coeliac disease targeted gliadin epitopes which are rendered immunogenic by deamidation of glutamine residues [19,22,23].

Deamidation of beta-cell antigens, and proinsulin specifically, have been suggested to create neoepitopes that are recognized by T1D-associated CD4^+^ T cells in people with T1D [3,24]. Beta cells are believed to be particularly susceptible to endoplasmic reticulum (ER) stress [25]. It has been suggested that ER stress, due to insulin demand and/or viral infection, may promote the activity of tissue transglutaminase (tTG) which in turn deamidates glutamine residues [24]. Van Lummel et al. tested an array of candidate epitopes derived from beta cell antigens. They found that deamidated PI_33-45_ (EAEDL**E**VG**E**VELG) bound more strongly to HLA-DQ8 and HLA-DQ8*trans* than the native sequence (EAEDL**Q**VG**Q**VELG) [26]. They also found peripheral blood responses were more frequently detected to deamidated B-chain-C-peptide (PI_30-43,_ TRREAEDL**E**VG**E**VE) peptide, than native PI_30-43_ peptide (TRREAEDL**Q**VG**Q**VE) in samples from subjects with recent onset T1D [26]. This led to the suggestion that CD4^+^ T-cell responses to *deamidated* C-peptide initiated anti-beta cell autoimmunity which then spread to unmodified C-peptide. More recently, exposure of human islets, *in vitro*, to inflammatory cytokines promoted the formation of deamidated C-peptide, suggesting a mechanistic link between T-cell infiltration/inflammatory cytokines and the deamidation of C-peptide and autoimmune T-cell responses [27]. Furthermore, a Non-Obese Diabetic (NOD) mouse study showed by mass spectrometry analysis of beta-cell granule proteins that deamidated C-peptide is present in the beta-cells of NOD mice that develop autoimmune diabetes [28].

Given the strong evidence for CD4^+^ T-cell responses against full-length C-peptide *and* the role of neoepitopes formed by deamidation in the immune pathogenesis of T1D, we asked the following question. Compared to unmodified C-peptide, does deamidated C-peptide stimulated stronger, or more frequent, CD4^+^ T-cell responses in PBMC of people with T1D? We took two approaches to answering this question. First, we analyzed the impact of glutamine deamidation on the responses of C-peptide specific T-cell lines that express TCRs derived from human islet-infiltrating CD4^+^ T cells. Second, we compared CD4^+^ T-cell responses against unmodified full-length C-peptide (PI_33-63_) to responses against variants of C-peptide comprising glutamine (Q) to glutamic acid (E) substitutions in PBMC from individuals with recent onset T1D. Neither approach provided any evidence that deamidated C-peptide was more immunogenic than unmodified C-peptide. Hence, we conclude that *deamidated* C-peptide, in contrast to unmodified C-peptide, is not an important target of autoimmune CD4^+^ T-cell responses in people who develop T1D.

## 2 Material and methods

### 2.1 Subjects

Ethical approval was given by St Vincent's Hospital (HREC-A 135/08), Royal Melbourne Hospital (2009.026) and Monash Health (12185B) ethics committees. All participants provided written informed consent. T1D was diagnosed according to American Diabetes Association criteria [29]. Details of all participants are shown in Supplementary Tables 1 and 2

### 2.2 Media and cell lines

Epstein Barr Virus (EBV) transformed B cell lines were used as antigen presenting cells (APC). Supplementary Table 3 summarizes the EBV lines used and their HLA class II genotypes. Jurkat E6-1 (CellBank Australia) [30,31] were modified by CRISPR/Cas9 to knock out the endogenous TCRA, TRB and CD4 genes. These cells were then transduced with lentivirus encoding human CD4. HEK293T (CellBank Australia) and other cell lines were cultured in RPMI-1640 supplemented with non-essential amino acids (Gibco), 2 mM Glutamax (Gibco), 100U/ml penicillin (Sigma), 0.1 mg/ml streptomycin (Sigma) and 5% or 10% foetal bovine serum (FBS) or pooled human serum.

### 2.3 Lentivirus production

Plasmids used for making lentiviruses are listed in Supplementary Table 4. For lentivirus-mediated gene transduction, genes to be expressed were cloned into modified versions of pRRLSIN.cPPT.PGK-GFP.WPRE. The EGFP gene was excised by *Bam*HI/SalI digestion and replaced by human TRAC or TRBC with either a PmeI (TRAC) or SbfI (TRBC) site at the 5′ end of the TCR constant regions. All TCR genes were synthesised by IDT as geneblocks and cloned into the modified pRRLSIN vectors by In-fusion cloning according to the manufacturer's (Takara) protocol. Plasmids were extracted from growing *E. coli* and the inserts were verified by Sanger sequencing. For transfection of HEK293T cells, TCR gene-containing plasmids were purified using Macherey-Nagel kits according to the manufacturer's recommendations. HEK293T cells were transfected with the appropriate pRRLSIN construct and the packaging plasmids: pMDLg/pRRE, pRSV-Rev, and pMD2.g using Lipofectamine 2000 (Invitrogen) as per manufacturer's instructions. After 7–8 h incubation at 37 °C, 5% CO_2_, the medium was changed. After a further 1–2.5 days incubation, supernatant was collected and filtered through a 0.45 μm low protein binding filter. Lentivirus-containing supernatant was either used fresh, or frozen at ^-^80^o^C until required. Supplementary Table 5 summarizes the Jurkat lines and their antigen specificity.

### 2.4 Lentiviral transductions

Jurkat cells were resuspended in viral supernatant at 1–2 x 10^6^/ml with 5 μg/ml polybrene. Cells were centrifuged at 1200 rpm (300×*g*) for 60min at room temperature, then diluted 1:1 in fresh medium and incubated overnight at 37 °C, 5% CO_2_. Cells were grown for a week; during this time, they were washed six times. Transduced cells were purified either by flow cytometry or magnetic bead enrichment, as described below.

### 2.5 Flow cytometry

Flow cytometry was performed on a Becton Dickinson LSR Fortessa. The following anti-human mAbs were used for FACS staining: CD3-PE (UCHT1, BD Biosciences) anti CD4-PE (RPA-T4, BD Biosciences) anti-TCRαβ-AF647 (IP26, Biolegend). Cells were stained with the appropriate mAb in PBS 0.1% FBS for 20 min on ice, then washed twice. Dead cells were excluded by propidium iodide staining. Compensation settings were determined using single color controls. TCR transduced Jurkat cells were purified by sorting viable (propidium iodide negative) CD3^+^/TCRαβ^+^ cells.

### 2.6 Purification of TCR-transduced cells

A REALease CD3 Microbead Kit (Miltenyi) was used to purify TCR-transduced Jurkat cells. Briefly, 3–5x10^6^ cells were labelled with REALease CD3-biotin followed by REALease anti-biotin MicroBeads. Labelled cells were applied to a MS MACS column on a magnetic stand. After washing through the unlabeled cells, the column was removed from the magnet and the CD3-expressing cells were eluted. Then the microbeads and the REALease Complex were removed. After washing in PBS, the purified cells were cultured for 6–7 days before purity was checked by staining for CD3 and TCRαβ (as above) and FACS analysis. In some experiments, the number of viable cells were counted by Trypan Blue exclusion using a hemocytometer at different times after purification.

### 2.7 T-cell activation

TCR-transduced T cells were cultured, in triplicate, in cRPMI/5% FBS at 2 × 10^4^/well with an equal number of EBV-transformed B cells (see Supplementary Table 3) and different peptides (Purar Chemicals). See Supplementary Table 6 for a full list of peptides used in this study. The positive control was PMA (10 ng/mL) and ionomycin (500 ng/mL) in culture media. Negative controls had all cells, but no peptide. After overnight culture supernatant was collected for IL-2 ELISA.

### 2.8 IL-2 ELISA

ELISAs were performed using the ELISA MAX™ Deluxe set Human IL-2 (BioLegend) as per the manufacturer's instructions. Briefly, 96-well ELISA plates (Greiner Bio-One) were coated with 100μL/well of purified anti-human IL-2 antibody diluted 1:200. The following day, the plate was washed three times in 0.05% Tween/PBS, then blocked with 200μL/well of 1%BSA/PBS at room temperature for 1 h. The Jurkat supernatant (100 μL) was transferred to the ELISA plates. An IL-2 standard was prepared by two-fold serial dilutions of 1,000 pg/mL recombinant IL-2. were prepared and added, in triplicates, to the mAb coated ELISA plate. After adding detection reagents, the plate was washed and 100μL/well of 3,3′,5,5′-Tetramethylbenzidine (TMB) solution was added. The reaction was stopped by the addition of 2 M H_2_SO_4_ solution (BioLegend) and optical density (OD) measured using a FLUOstar Omega microplate reader (BMG LabTech). The amount of IL-2 secreted was determined using a linear regression of all OD values (minus the background) of the standard curve. The maximum response was defined as the amount of IL-2 secreted by the PMA/Ionomycin stimulated Jurkat T cells. The 50% effective concentration (EC_50_) was calculated and the fold change of the amount of IL-2 secreted in response to the deamidated peptides relative to the amount secreted in response to native C-peptide was determined.

### 2.9 CFSE-based proliferation assay

Blood was obtained by venepuncture. Peripheral blood mononuclear cells (PBMC) were isolated over Ficoll-paque (GE Healthcare) and washed twice in phosphate buffered saline (PBS) as described [32]. The CFSE (5,6-carboxyfluorescein diacetate succinimidyl ester) proliferation assays were performed as described previously [16,32]. CFSE-labelled PBMC were cultured with either: no antigen, full-length C-peptide (^PI^33-63), or ^PI^33-63 Q38E (referred to as Q6E), ^PI^33-63 Q41E (referred to as Q9E), ^PI^33-63 Q54E, (referred to as Q22E) ^PI^33-63 Q63E (referred to as Q31E), or ^PI^33-63 with all glutamines substituted for glutamic acid, (referred to as QallE). See Supplementary Table 5 for a detailed description of the peptides. Each peptide was used at a final concentration of 10 μM. Positive controls were tetanus toxoid (TT 10LfU/ml, Statens Serum Institut) and/or anti-CD3 (OKT3 10 ng/ml). The results are presented as a cell division index (CDI), the ratio of the number of CD4^+^ T cells that have proliferated in the presence of antigen to the number of CD4^+^ T cells that have proliferated in the absence of antigen [32,33].

### 2.10 Statistical analysis

Prism 9.4.1 was used to analyze the data and perform statistical analysis. Comparisons between group data were made using a two tailed Mann-Whitney test. Data from CFSE-based proliferation assays was log_10_ transformed before using the Wilcoxon matched-pairs signed rank test. Statistical significance was defined as p < 0.05.

## 3 Results

### 3.1 Generation of C-peptide specific Jurkat lines

CRISPR/Cas9 was used to knock out the endogenous TRAC, TRBC and CD4 genes in the human T-cell line Jurkat. This gave us a recipient cell line to which CD4 or CD8αβ was introduced by lentiviral transduction. Here we used TCR^−/−^ Jurkat cells that expressed human CD4 and transduced them with lentiviruses to express the TCRαβ genes of the TCRs derived from a panel of human CD4^+^ T-cell clones specific for epitopes derived from human proinsulin C-peptide (PI_33-63,_ referred to here as wild type (WT) C-peptide). Six of these clones were isolated from CD4^+^ T cells that infiltrated the pancreatic islets of an organ donor who suffered from T1D [7], and 12 from the peripheral blood of six people with T1D [12]. Each of the 18 TCR transduced Jurkat lines expressed a different C-peptide specific TCR. The minimum epitope and HLA restriction of each clone was determined previously [7,12] and these data are summarized in Supplementary Table 4.

### 3.2 Validating the Jurkat-based assays

**Fig. 1:**
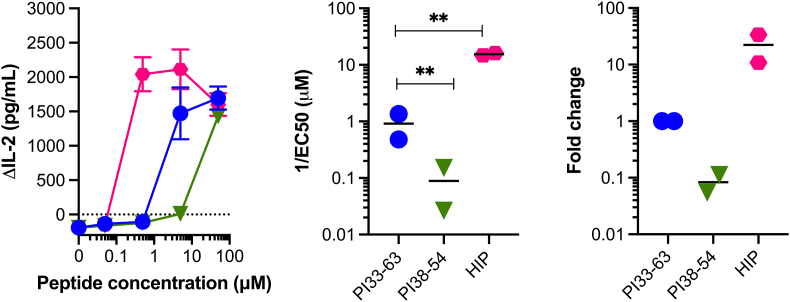
Detection of differences in peptide potency using Jurkat lines Jurkat cells expressing a the TCR from the islet-infiltrating CD4^+^ T-cell clone, A3.10. For all panels the blue circles are full-length WT C-peptide (PI33-63), the teal triangles short C-peptide (PI38-54) and the pink hexagons are responses to HIP (Cpept-IAPP2 HIP) known to be a potent stimulator of this clone (see Supplementary Table 4 for sequences). (A) IL-2- peptide dose response curve with a CD4^+^ Jurkat cells expressing the A3.10 TCR. Mean of triplicate measure and bars are the SEM. (B) shows the inverse of the EC50 for the titration shown in panel A. Unpaired *t*-test (** = p < 0.01). (C) Fold change in EC50 relative to full-length C-peptide (PI33-63). For panels B and C each point represents an independent experiment.

To validate our assay, we used a Jurkat line that expressed the TCR from the human islet-infiltrating CD4^+^ T-cell clone ACD4_2. Jurkat cells produce abundant IL-2 when TCR signaling is activated, but no IL-2 is produced without TCR activation. Jurkat cells also produce IL-8 [34], but we found this was secreted at lower concentrations than IL-2. Hence, we used secretion of IL-2 as a readout of antigen recognition. This clone responds very sensitively to a Cpeptide-IAPP2 hybrid insulin peptide (HIP) [35]. It can also respond to C-peptide which comprises half of the HIP. It responds moderately to full-length C-peptide (PI_33-63_), but weakly to an 18mer fragment of C-peptide (PI_38-54_) [36]. This allowed us to validate our system with a set of peptides known to vary in their capacity to stimulate this clone. Secretion of IL-2 into the culture media served as a readout of TCR recognition of peptide and the relative capacity of the same Jurkat line to respond to different peptides was determined by peptide titration (Fig. 1). There was a clear difference in the sensitivity of the line to each peptide measured by IL-2 secretion in response to graded doses of each peptide. The HIP was a 22.3-fold more potent agonist than full-length C-peptide, whereas the short C-peptide was 0.1 times as potent as full-length C-peptide. Hence, differences in the agonist capacity of peptide variants could be defined using TCR transduced Jurkat lines.

### 3.3 Assessing the sensitivity of primary T-cell derived TCRs to deamidated C-peptide

**Fig. 2:**
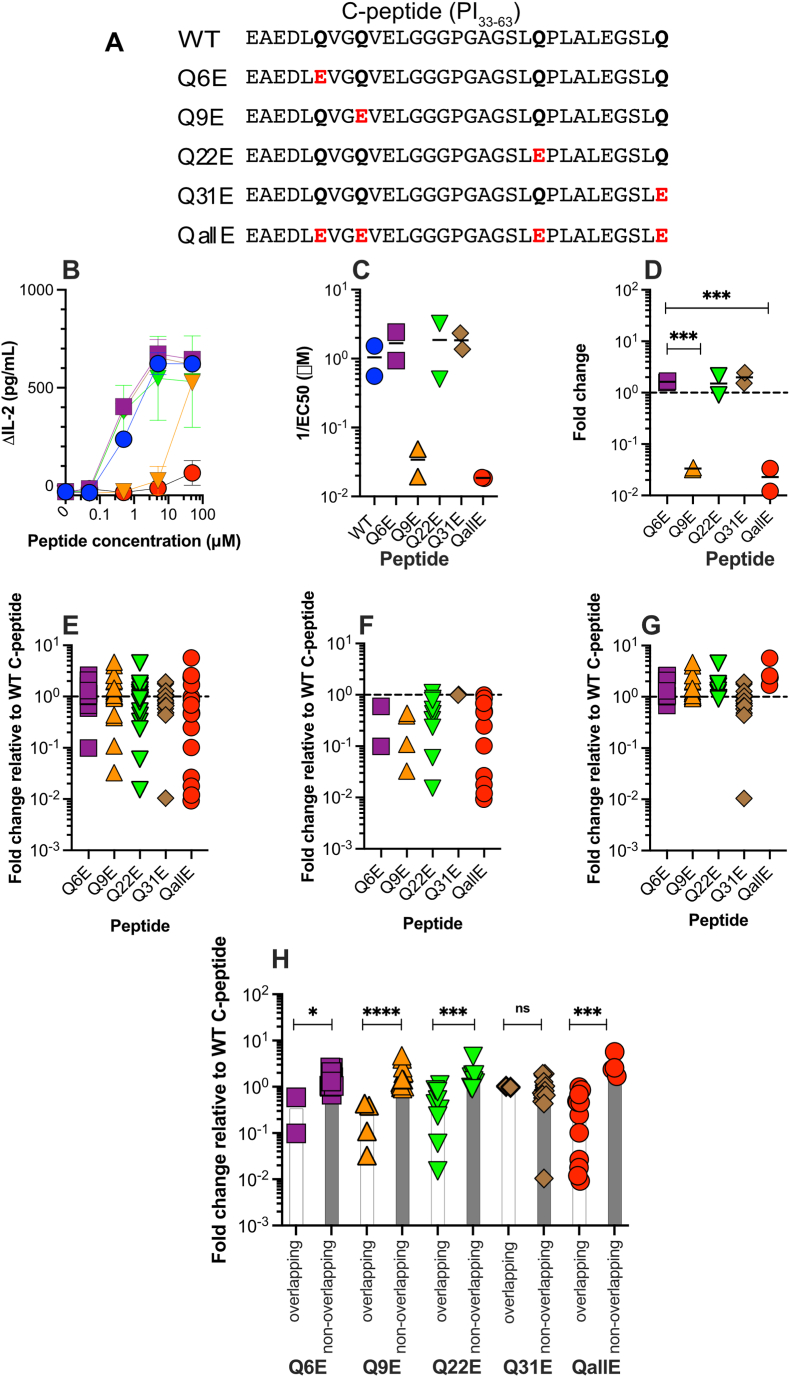
*C-peptide specific TCR responses to deamidated C-peptide variants* (A) Each peptide is referred to by the position, within C-peptide of the glutamine (Q), in bold, to glutamic acid (E), in red text, substitutions. The native sequence is referred to as wildtype (WT) C-peptide. The five variants are named according to the position within C-peptide of the substitution (i.e Q6E, Q9E etc), except for the peptide with all Qs substituted for E (QallE). (B) An example of a titration experiment using a Jurkat line (ACD4_38) expressing a C-peptide specific TCR. Each point represents the mean of triplicate measures and vertical bars, the standard deviation. (C) The EC_50_ for each peptide was calculated the and the EC_50_ from two independent experiments are shown. The bars represent the mean of the two experiments. (D) The fold change in EC_50_, relative to WT C-peptide for this TCR. The dotted line indicates a fold change of 1 relative to WT C-peptide. (E) A summary of the fold change, relative to WT C-peptide for all 18 clones tested. (F). Fold change for clones where the Q to E substitution falls within (F), or outside (G) the mapped epitope. (H) A comparison of the responses to a peptide when the modification falls within (overlapping) or outside (non-overlapping) the mapped epitope. Statistical significance was determined using the Mann-Whitney test (ns, not significant, 8, p < 0.05, **p < 0.01, ***p < 0.001).

To determine if C-peptide specific TCRs were more sensitive to Q to E variants of C-peptide we tested graded concentrations of wild type C-peptide and five deamidated variants. Four variants had a single naturally occurring glutamine substituted for glutamic acid (Q6E, Q9E, Q22E, Q31E) and one peptide had all four glutamines substituted for glutamic acid (QallE) (Fig. 2A). The results from a peptide induced IL-2 production experiment with clone ACD4_38 are shown in Fig. 2B. Titration curves were used to determine the effective concentration for 50% response (EC_50_). The EC_50_ for each peptide in two independent experiments is shown in Fig. 2C. To compare the relative potency of the Q to E substituted peptides the responses were calculated as fold change relative to full-length, WT C-peptide (Fig. 2D). For this clone peptide Q9E and QallE were dramatically less stimulatory than WT C-peptide and the other single substituted peptides. The epitope recognized by ACD4_38 is QVELGGPGA [12] (PI_41-50_). The first residue of this epitope is Q9, which when substituted for E dramatically diminishes T-cell recognition.

Collectively when all 18 Jurkat T-cell line responses are considered, Q to E substitution diminishes T-cell responsiveness (Fig. 2E). In some cases, the EC_50_ dropped 10–100 fold. Because the epitopes were known for these clones, we separated the clones which had a substitution within the cognate epitope from those where the substitution fell outside the epitope. In all cases, when the Q to E substitution fell within the epitope, the fold change in EC_50_ relative to WT C-peptide decreased (Fig. 2F). In contrast, the potency of peptides with substitutions falling outside of the mapped epitope was not decreased. In many cases the potency of the peptides was slightly increased, up to 5-fold more than WT C-peptide (Fig. 2G). To investigate this further we compared the fold change in EC_50_ between clones that recognized epitopes containing a substitution (labelled ‘overlapping’) to the remaining clones whose epitope did not contain a substitution (labelled ‘non-overlapping’, Fig. 2H). This analysis showed that for Q6E, Q9E, Q22E and QallE, there was a statistically significant difference in the responses, relative to full-length C-peptide. The exception was Q31E, for which there was no difference. Titration curves for all Jurkat lines are shown in Supplementary Fig. 1.

### 3.4 Analysis of polyclonal T-cell responses to deamidated C-peptide in PBMC

**Fig. 3:**
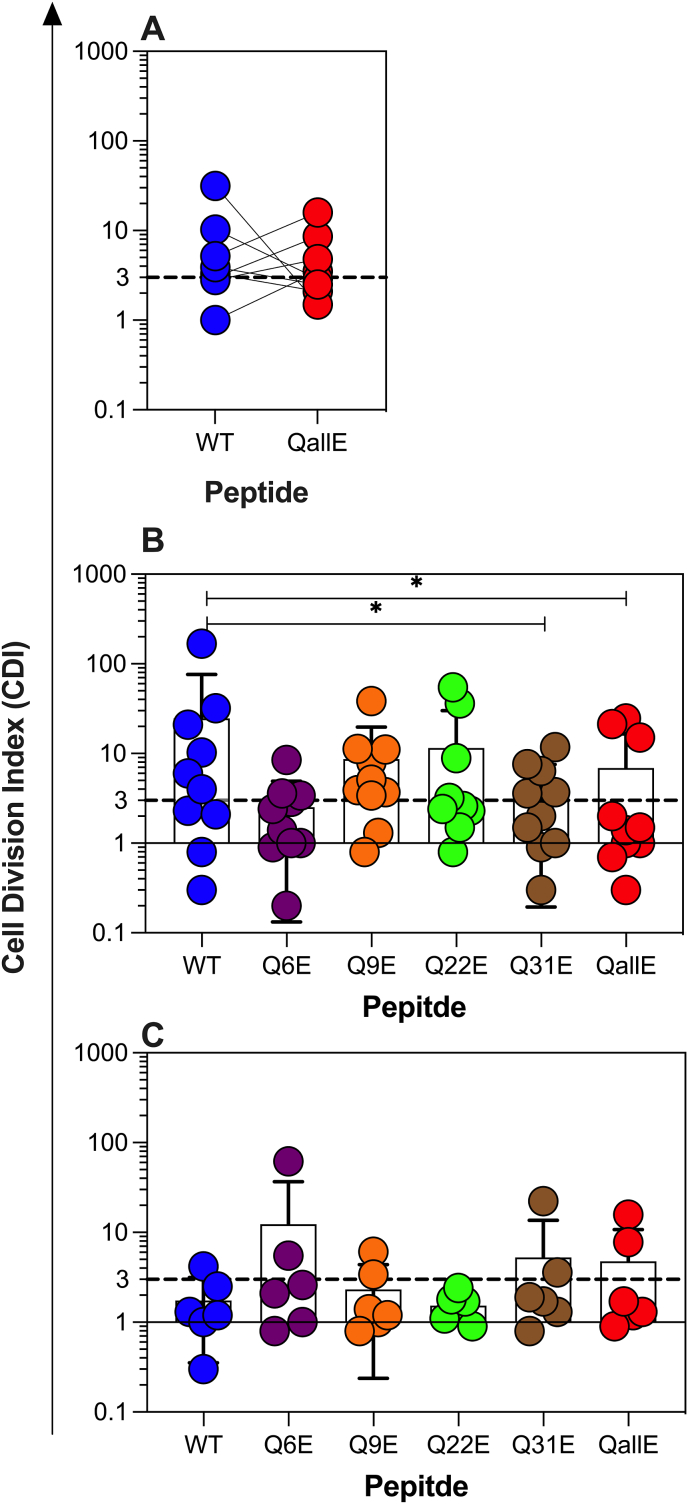
PBMC responses to WT and deamidated C-peptide variants. (A) Proliferation in responses, measured as a cell division index (CDI) to WT or QallE C-peptide were compared in 8 PBMC from 8 people with recent onset T1D. Responses from the same individual are linked. There was no statistically significant difference between the groups. (B) PBMC proliferation responses to WT and C-peptide variants with individual Q to E substitution and QallE from people with recent onset T1D (B) and HLA matched control subjects who don't have T1D (C). Only difference which reached statistical significance are marked (* = P < 0.05). For responses in subjects without T1D, none of the differences between groups reached statistical significance, p > 0.05, by Wilcoxon matched pairs signed rank test of Log 10 transformed CDI data. Each point is a different individual, bars show the mean and the standard deviation for each group.

Our analysis of CD4^+^ Jurkat lines expressing TCRs derived from primary CD4^+^ T cells used TCRs derived from CD4^+^ T cells that were selected for their specificity for WT C-peptide [7,12]. Our data suggested that Q to E substitution at best modestly enhanced recognition, but in most cases attenuated responses. This may be due to Q to E substitutions being poorly tolerated by the TCRs that were selected based on their recognition of WT C-peptide. To address this, we measured polyclonal CD4^+^ T-cell responses in PBMC from people with recent onset type 1 diabetes. (see Supplementary Tables 5 and 6 for details of each subject). When we compared responses to WT C-peptide and QallE using the CFSE-based proliferation assay [32], we found that QallE stimulated a slightly weaker response than WT C-peptide (Fig. 3A), although in some individuals there was a modest increase in the response. Next, in a separate cohort we investigated CD4^+^ T-cell responses to the five C-peptide variants with individual Q to E substitutions in PBMC of people with recent onset type 1 diabetes (Fig. 3B). We found, as expected [12], that a majority of PBMC samples responded to WT C-peptide, but responses to Q to E substituted C-peptide variants were not stronger, in fact in most cases they were weaker, than responses to WT C-peptide. (See Supplementary Tables 7–9 for results from individual subjects). Finally, we asked if people without T1D, but who carried the T1D-associated HLA alleles, HLA-DQ2, or HLA-DQ8 had stronger responses to Q to E substituted C-peptide variants (Fig. 3C). Here few subjects had response to WT C-peptide and only a minority had greater responses to Q to E substituted variants of C-peptide, compared to WT C-peptide. This indicates that deamidation of C-peptide does not create a neoepitope that stimulates CD4^+^ T-cell proliferation in people without type 1 diabetes.

## 4 Discussion

Here we show that full length C-peptide comprising glutamic acid, in place of glutamine, does not increase the immunogenicity of full-length C-peptide (PI_33-63_). Our findings do not support the notion that deamidated C-peptide primes CD4^+^ T-cell responses in people with T1D [26,27]. We reached this conclusion using two approaches. First, we compared the capacity of deamidated and native C-peptide to stimulate a large panel of 18 Jurkat T-cell lines that express different TCRs derived from CD4^+^ T-cell clones specific for C-peptide. These CD4^+^ T cells were isolated from human islet-infiltrating CD4^+^ T cells of a cadaveric organ donor [7], and from the peripheral blood of people with recent onset T1D [12]. In both studies, these CD4^+^ T cells were all selected based on their capacity to recognize WT C-peptide.

Why do our results differ from earlier work which reached the conclusion that deamidated C-peptide is a triggering antigen in human T1D? There are several technical differences in our approach compared to van Lummel et al. [26]. For example, they measured responses to 16mer (PI_30-43_) in PBMC using ELISpot for IFNγ and IL-10 [26], whereas we analyzed CD4^+^ T-cell proliferation in responses to full-length C-peptide using the CFSE-based proliferation assay [32]. The IFNγ ELIspot responses reported by van Lummel et al. [26] were rarely detected and when detected they were weak; ∼4–5 fold background, with a cut of 3-fold background.

The strength of our approach is that it allowed us to measure the relative potency of WT full-length C-peptide and compare it directly to peptides with individual Q to E substitutions. In all cases the epitope recognized by each clone had been mapped [7,12]. This allowed us to separate Q to E substitutions that fell within the epitope recognized by the TCR from those that fell outside the epitope. We found that substitutions that fell outside the epitope frequently increase potency of the peptide for stimulating TCR recognition. The increase in substituted peptide potency, relative to WT C-peptide, was always modest. Currently it is not clear how a substitution outside the epitope can augment T-cell recognition. We did not observe any clear patterns in distance from the ‘core epitope’ to the substituted amino acid, although the number of clones may be too small to pick up a subtle effect. Our earlier work highlighted how a longer peptide is a more potent agonist than shorter peptides for some C-peptide specific clones [12]. Our data here supports the notion that ‘flanking’ residues can modulate T-cell recognition of C-peptide (reviewed by Ref. [37]). Nonetheless, our T-cell clone based functional analysis does not support the notion that neoepitopes formed by the conversion of glutamine to glutamic acid are major epitopes in human T1D.

An important caveat from our T-cell clone analysis is that the clones were selected based on their capacity to respond to WT C-peptide. It may not be surprising then that these clones are not potently stimulated by C-peptide variants. To address this possibility, we investigated the capacity of C-peptide, incorporating different Q to E substitutions to stimulate CD4^+^ T-cell responses in the PBMC of people with and without T1D. Using a polyclonal population and the highly sensitive CFSE-based proliferation assay [32] allowed us to detect responses to WT C-peptide, at a similar frequency to that seen in our earlier work [12], and compare them to responses to Q to E substituted C-peptide variants. We found no evidence for an augmented response to Q to E substituted C-peptide. On an individual basis there were occasional subject where a modest increase in response was seen to a Q to E substituted peptide, so we cannot exclude that in some individuals these peptides stimulate greater responses. However, the increase in responses, if seen, are modest and no single Q to E substituted peptide is a more potent agonist than the others in these assays.

Our study has some limitations. First, we have not attempted to clone and analyze the specificity of CD4^+^ T cells that responded to deamidated C-peptide. It remains unknow if the responses to these peptides are due to the Q to E substitution, or if the unmodified region of the peptide is being recognized by the responding CD4^+^ T cells. Nonetheless, it is clear at a polyclonal level that C-peptide variants that contain Q to E substitutions are not more immunogenic than unmodified C-peptide. Second, our conclusions only apply to C-peptide. We have not examined other parts of insulin, nor other beta-cell antigens, hence we cannot comment on the putative immunogenicity of other beta-cell proteins upon deamidation.

## 5 Conclusion

In conclusion, functional analysis of C-peptide specific TCRs and analysis of peripheral blood from people with recent onset T1D showed that substitution of glutamine with glutamic acid does not increase the immunogenicity of C-peptide. We conclude that deamidation of C-peptide does not play a direct role in the autoimmune beta-cell destruction in type 1 diabetes.

## Author contributions

**Abby Foster**: Investigation, writing-Review and editing, formal analysis; **Pushpak Bhattacharjee**: Investigation, Project administration; **Miha Pakusch**: Investigation, Project administration; **Eleonora Tresoldi**: Investigation, Project administration; **Fergus Cameron**: Resources; Project administration; **Stuart Mannering**: Conceptualization, Writing-original draft, Visualization, Supervision, formal analysis, funding acquisition.

## Declaration of competing interest

The authors declare that they have no known competing financial interests or personal relationships that could have appeared to influence the work reported in this paper.

## Data Availability

Data will be made available on request.
