# Supplementary material for: Glutamine deamidation does not increase the immunogenicity of C-peptide in people with type 1 diabetes
Source: J Transl Autoimmun. 2022 Dec 27;6:100180. doi: 10.1016/j.jtauto.2022.100180 (PMC9811213; doi:10.1016/j.jtauto.2022.100180)
Supplement: Multimedia component 1 [file mmc1.docx]

**Supplementary Materials**

for

**Glutamine Deamidation does not increase the immunogenicity of C-peptide in people with type 1 diabetes**

by

Abby Foster, Pushpak Bhattacharjee, Miha Pakusch, Eleonora Tresoldi, Fergus J. Cameron, Stuart I. Mannering

**Supplementary Materials**

**Supplementary Table 1** Characteristics of subjects with T1D

| **Donor** | **Age (years)** | **Sex** | **Time since Dx** | **HLA** | | |
| --- | --- | --- | --- | --- | --- | --- |
|  |  |  |  | **-DR** | **-DQ** | **-DP** |
| **1** | 12 | M | 4y | DRB1*04:05, 16:01; DRB4*01:03; DRB5*02:02 | DQB1*02:02, 05:02; DQA1*01:02, 03:03 | DPB1*02:01, 04:01; DPA1*01:03; 02:01 |
| **2** | 9 | F | 1 d | DRB1*03:01:01G, 07:01:01G | DQB1*02:01:01G | NA |
| **3** | 17 | F | 1 d | DRB1*03:01, 07:01 DRB3*02:02 DRB4*01:03 | DQB1*02:01, 02:02 DQA1*02:01, 05:01 | DPB1*04:03, 13:01 DPA1*01:03, 02:01 |
| **4** | 9 | M | 1 d | DRB1*04:01, 04:04; DRB4*01:03 | DQB1*03:02; DQA1*03:01 | DPB1*04:01; DPA1*01:03 |
| **5** | 13 | M | 1 d | DRB1*04:01, 08:04 DRB4*01:03 | DQB1*03:02, 04:02 DQA1*03:01, 04:01 | DPB1*04:01, 104:01 DPA1*01:03 |
| **6** | 2 | M | 1 d | NT | NT | NT |
| **7** | 5 | M | 1 m | DRB1*03:01, 04:05 DRB3*01:01 DRB4*01:03 | DQB1*02:01/163N, 03:02/289 DQA1*03:03, 05, 01 | DPB1*04:01, 124:01 DPA1*01:03 |
| **8** | 22 | F | 13 y | DRB1*03:01, 04:01 DRB3*02:02 DRB4*01:03 | DQB1*02:01, 03:02  DQA1*03:01, 05:01 | DPB1*02:02, 03:01 DPA1*03:01, 05:01 |
| **9** | 15 | M | 1 m | DRB1*01:01. 15:02 DRB5*01:02 | DQB1*05:01, 06:01 DQA1*01:01, 01:03 | DPB1*04:01, 13:01 |
| **10** | 10 | F | 3 m | DRB1*01:01:01, 04:01:01; DRB4*01:03:01 | DQB1*03:01:01G, 05:01:01G; DQA1*01:01:01, 03:03:01 | DPB1*02:01:02, 04:01:01; DPA1*01:03:01 |
| **11** | 10 | M | 3 m | DRB1*03:01:01; DRB3*02:02:01 | DQB1*02:01:1G; DQA1*05:01:01 | DPB1*04:01:01, 104:01:01; DPA1*01:03:01 |
| **12** | 10 | F | 2 m | DRB1*04:01:01, 13:02:01; BRB3*03:01:01; DRB4*01:03:01 | DQB1*03:01:01G, 06:04:01; DQA1*01:02:01, 01:03:01 | DPB1*03:01:01, 04:01:01; DPA1*01:03:01 |
| **13** | 14 | M | 1 m | DRB1*03:01:01; DRB3*01:01:02, 02:02:01 | DQB1*02:02:01G; DQA1*05:01:01 | DPB1*03:01:01, 04:01:01; DQA1*05:01:01 |
| **14** | 12 | M | 1 d | DRB1*04:01, 07:01 DRB4*01:01, 01:03 | DQB1*02:02, 03:01 DQA1*02:01, 03:03 | DPB1*04:01, 11:01 DPA1*01:03, 02:01 |
| **15** | 12 | M | 1 d | DRB1*04:04; 07:01 DRB4*01:03 | DQB1*  02:02; 03:02  DQA1*  02:01; 03:01 | DPB1*04:02 DPA1*01:03 |
| **16** | 2 | M | 2 m | DRB1*13:01; 16:01  DRB3*02:02  DRB5*02:02 | DQB1*02:01; 05:02  DQA1*01:02; 05:01 | DPB1*04:01; 105:01  DPA1*01:03; 03:01 |
| **17** | 9 | M | 2 m | DRB1*07:01; 09:01  DRB4*01:01; 01:03 | DQB1* 02:02; 03:03  DQA1*02:01l 03:02 | DPB1*11:01; 14:01  DPA1*02:01 |
| **18** | 10 | F | 2 m | NT | NT | NT |
| **19** | 10 | F | ND | DRB1*03:01 DRB3*01:01, 02:02 | DQB1*02:01 DQA1*05:01 | DPB1*03:01, 04:02 DPA1*01:03 |

NT: Not tested

ND: Not disclosed

Y, year; m, month; d, day

**Supplementary Table 2.** Summary of subjects without T1D

| **Donor** | **Age** | **Sex** | **HLA^#^** | |
| --- | --- | --- | --- | --- |
|  |  |  | **-DR** | **-DQ** |
| **1** | 49 | F | DRB1*04:01, 14:03 | DQB1*03:01:01G, 03:02:01G |
| **2** | 39 | F | DRB1*04:03, 14:01:01G | DQB1*03:02:01G, 05:02:01G |
| **3** | 44 | M | DRB1*04:01, 04:08 | DQB1*03:01, 03:02; DQA1*03:01/02/03 |
| **4** | ND | F | DRB1*03, 04 | DQB1*02:01/14, 03:01; DQA1*03:02/03, 05:01 |
| **5** | 41 | F | DRB1*04:01, 15:01:01G | DQB1*03:02:01G, 06:02:01G |
| **6** | 54 | F | DRB1*02, 04 | DQB1*03:02, 06:02; DQA1*01:02, 03:01/02/03 |

NT: not tested

ND: Not disclosed

^#^HLA-DP alleles were not determined

**Supplementary Table 3.** EBV transformed B-cell lines used in the study

| **Name** | **HLA Class II** | | |
| --- | --- | --- | --- |
|  | **HLA-DR** | **HLA-DQ** | **HLA-DP** |
| **EBV-9031** | DRB1*04:01:01; -  DRB4*01:03:01:01;- | DQA1*03:01:01;-  DQB1*03:02:01,- | DPA1*01:03:01;-  DPB1*04:01:01;- |
| **EBV-9022** | DRB1*03:01;-  DRB3*01:01;- | DQA1*05:01:01;-  DQB1*02:01;- | DPA1*01:03:01;-  DPB1*03:01:01;- |
| **EBV-KJ** | DRB1*03:01;  DRB1*04:04 | DQA1*03:01:01;  DQA1*05:01:01;  DQB1*02:01;  DQB1*03:02; | ND |

ND; Not determined

**Supplementary Table 4**. Plasmids used for generating lentivirus

| **Plasmid name** | **Protein encoded** | **Source** |
| --- | --- | --- |
| pRRLSIN.cPPT.PGK-GFP.WPRE | EGFP | A gift from Didier Trono (Addgene plasmid # 12252 ; http://n2t.net/addgene:12252 ; RRID:Addgene_12252). |
| pMDLg/pRRE | HIV Gag, Pol | A gift from Didier Trono (Addgene plasmid # 12251; http://n2t.net/addgene:12251; RRID:Addgene_12251). |
| pRSV-Rev | Rev | A gift from Didier Trono (Addgene plasmid # 12253; http://n2t.net/addgene:12253 ; RRID:Addgene_12253). |
| pMD2.G | VSV-G | A gift from Didier Trono (Addgene plasmid # 12259; http://n2t.net/addgene:12259 ; RRID:Addgene_12259). |
| pRRLSIN.cPPT.PGK-TRAC.WPRE | TCR alpha constant region | Prepared in house |
| pRRLSIN.cPPT.PGK-TRBC.WPRE | TCR beta 2 constant region | Prepared in house |

**Supplementary Table 5.** Summary of TCR-transduced Jurkat lines

| **Name** | **HLA-restriction** | **Epitope** | **Source** | **Reference** |
| --- | --- | --- | --- | --- |
| ACD4_1 | DQ8 | AGSLQPLALE | Islets | [1] |
| ACD4_9 | DQ8 | AGSLQPLAL | Islets | [1] |
| ACD4_2 | DQ8 | VELGGGPGA | Islets | [1] |
| ACD4_7 | DQ8 | VELGGGPGA | Islets | [1] |
| ACD4_38 | DQ8 | QVELGGGPG | Islets | [1] |
| ACD4_8 | DQ8 | VELGGGPGA | Islets | [1] |
| A3.10 | DQ8 | VELGGGNAVEVLK | Islets | [1-3] |
| BeHeCD4_24 | DQ8 | PGAGSLQPLALE | PBMC | [4] |
| BeHeCD4_9 | DR4 | SLQPLALEGSL | PBMC | [4] |
| KaMaCD4_15 | DQ8*trans* | GAGSLQPLAL | PBMC | [4] |
| KaMaCD4_25 | DR4 | QPLALEGSLQ | PBMC | [4] |
| KaMaCD4_16 | DQ2 | QPLALEGSL | PBMC | [4] |
| KaMaCD4_4 | DQ8 | QVELGGGPGAG | PBMC | [4] |
| DeRyCD4_5 | DQ8 | AEDLQVGQV | PBMC | [4] |
| TeMcCD4_21 | DQ2/DQ2*trans* | SLQPLALEGSLQ | PBMC | [4] |
| HoMaCD4_22 | DQ8 | AGSLQPLAL | PBMC | [4] |
| HoMaCD4_14 | DQ8 | VELGGGPGAG | PBMC | [4] |
| EdHaCD4_6 | DQ2 | QPLALEGSL | PBMC | [4] |
| EdHaCD4_10 | DQ8 | EDLQVGQVELGG | PBMC | [4] |

**Supplementary Table 6.** Peptides used in this study.

| **Peptide Name** | **Amino acid Sequence** | **Mr (Da)** | **Purity (%)** |
| --- | --- | --- | --- |
| PI_33-63_ (WT) | EAEDLQVGQVELGGGPGAGSLQPLALEGSLQ | 3,020.3 | 93.1 |
| PI_33-63_ Q6E | EAEDL**E**VGQVELGGGPGAGSLQPLALEGSLQ | 3,021.2 | 90.9 |
| PI_33-63_ Q9E | EAEDLQVG**E**VELGGGPGAGSLQPLALEGSLQ | 3,021.2 | 93.9 |
| PI_33-63_ Q22E | EAEDLQVGQVELGGGPGAGSL**E**PLALEGSLQ | 3,021.2 | 94.9 |
| PI_33-63_ Q31E | EAEDLQVGQVELGGGPGAGSLQPLALEGSL**E** | 3,021.2 | 95.1 |
| PI_33-63_ QallE | EAEDL**E**VG**E**VELGGGPGAGSL**E**PLALEGSL**E** | 3,024.2 | 90.3 |
| HIP PI_40-47_/IAPP_52-58_ | GQVELGGGNAVEVLK | 1,469.7 | 89.5 |
| Short C-peptide  (PI_38-54_) | QVGQVELGGGPGAGSLQ | 1,553.7 | 92.7 |

**Supplementary RESULTS**

**Supplementary Table 7.** Individual data for Figure 2A: Mean CDIs from CSFE assay *with T1D* PBMC with WT or QallE peptide

| **Donor No** | **CDI WT** | **CDI Qall E** |
| --- | --- | --- |
| **1** | 1.0 | 3.5 |
| **2** | 3.0 | 8.6 |
| **3** | 3.3 | 2.1 |
| **4** | 10.3 | 2.9 |
| **5** | 2.8 | 4.8 |
| **6** | 31.6 | 1.5 |
| **7** | 3.9 | 2.5 |
| **8** | 5.2 | 15.9 |

**Supplementary Table 8.** Individual data for Figure 2B: Mean CDIs from CSFE assay *with T1D* PBMC with WT individually deamidated peptides

| **Donor No** | **CDI WT** | **Q6E** | **Q9E** | **Q22E** | **Q31E** | **CDI Qall E** |
| --- | --- | --- | --- | --- | --- | --- |
| **9** | 0.3 | 1.0 | 3.9 | 2.1 | 0.3 | 0.3 |
| **10** | 2.3 | 2.9 | 1.3 | 2.3 | 2.0 | 1.0 |
| **11** | 0.8 | 0.9 | 0.8 | 0.8 | 0.9 | 1.0 |
| **12** | 21.0 | 3.4 | 8.0 | 36.3 | 3.7 | 1.4 |
| **13** | 168.1 | 1.4 | 38.4 | 54.8 | 11.8 | 24.7 |
| **14** | 32.1 | 1.0 | 11.1 | 2.6 | 3.6 | 15.1 |
| **15** | 10.3 | 3.6 | 5.1 | 8.9 | 6.4 | 21.3 |
| **16** | 2.1 | 2.4 | 3.7 | 3.2 | 1.5 | 0.7 |
| **17** | 6.0 | 8.5 | 11.3 | 1.5 | 7.6 | 2.0 |
| **18** | 4.0 | 0.0 | 3.4 | 2.4 | 1.0 | 1.5 |

**Supplementary Table 9.** Individual data for Figure 2C: Mean CDIs from CSFE assay with PBMC from subjects without T1D, WT and individually deamidated peptides

| **Donor No** | **CDI WT** | **Q6E** | **Q9E** | **Q22E** | **Q31E** | **CDI Qall E** |
| --- | --- | --- | --- | --- | --- | --- |
| **1** | 1.3 | 1.0 | 1.0 | 1.3 | 1.3 | 15.8 |
| **2** | 1.0 | 2.6 | 1.4 | 1.1 | 1.7 | 1.2 |
| **3** | 1.2 | 2.1 | 1.2 | 0.9 | 3.6 | 0.9 |
| **4** | 0.3 | 0.8 | 0.8 | 1.7 | 0.8 | 1.3 |
| **5** | 4.2 | 62.1 | 6.1 | 1.8 | 22.3 | 1.7 |
| **6** | 2.5 | 5.5 | 3.4 | 2.4 | 1.9 | 7.8 |

**Supplementary Figure 1.** Dose response curves for C-peptide specific Jurkat lines against deamidated C-peptide variants.

**Supplementary Figure 1 cont.** Dose response curves for C-peptide specific Jurkat lines against deamidated C-peptide variants- continued.

**Legend Supplementary Figure 1.** Dose-response curves of Jurkat lines expressing C-peptide specific TCRs. Each line represents a different variant of C-peptide. Blue is unmodified C-peptide, purple squares are Q6E, orange triangles are Q9E, green triangles are Q22E, brown diamonds are Q31E and QallE is red circles. The IL-2 produced in the presence of antigen presenting cells and the indicated concentrations of peptide, minus the IL-2 produced in the absence of peptide, are shown. Each point is the mean of triplicate measures and the bars indicated the standard error of the mean. P/I is PMA/ionomycin, which is the positive control for Jurkat activation and IL-2 secretion. These data were used to calculate the EC_50_ and fold change in dose-response shown in Figure 1.

**SUPPLEMENTARY REFERENCES**

[1] V. Pathiraja, J. P. Kuehlich, P. D. Campbell, B. Krishnamurthy, T. Loudovaris, P. T. Coates *et al.* Proinsulin-specific, HLA-DQ8, and HLA-DQ8-transdimer-restricted CD4+ T cells infiltrate islets in type 1 diabetes. Diabetes, 2015;64**:**172-82.

[2] T. Delong, T. A. Wiles, R. L. Baker, B. Bradley, G. Barbour, R. Reisdorph *et al.* Pathogenic CD4 T cells in type 1 diabetes recognize epitopes formed by peptide fusion. Science, 2016;351**:**711-4.

[3] M. T. Tran, P. Faridi, J. J. Lim, Y. T. Ting, G. Onwukwe, P. Bhattacharjee *et al.* T cell receptor recognition of hybrid insulin peptides bound to HLA-DQ8. Nature communications, 2021;12**:**5110.

[4] M. So, C. M. Elso, E. Tresoldi, M. Pakusch, V. Pathiraja, J. M. Wentworth *et al.* Proinsulin C-peptide is an autoantigen in people with type 1 diabetes. Proceedings of the National Academy of Sciences of the United States of America, 2018;115**:**10732-7.
